# Supplementary material for: Development of a family-centered intervention to support self-determination in adolescents and young adults with intellectual disability in home environments: Protocol for a multistage mixed methods design
Source: PLoS One. 2025 Sep 4;20(9):e0325919. doi: 10.1371/journal.pone.0325919 (PMC12410785; doi:10.1371/journal.pone.0325919)
Supplement: Attachment — Submitted filename: Response to Reviewers.docx [file pone.0325919.s001.docx]

Hina Hadayat Ali, PhD

Academic Editor, PLOS ONE

Dear Dr. Ali,

We would like to thank you and the reviewers for your feedback on our manuscript entitled, “Development of a family-centered intervention to support self-determination in adolescents and young adults with intellectual disability in home environments: Protocol for a multistage mixed methods design” and for the opportunity to submit a revised version of the manuscript. As requested, we have revised the manuscript based on your and the reviewers’ suggestions, and we believe that these revisions have improved the overall quality of the manuscript. To facilitate review, we have created a table detailing our revisions, with our line-numbered-responses to each reviewer and every comment. We have also highlighted in yellow the changes made in the manuscript.

| **EDITOR** | | |
| --- | --- | --- |
|  | **Comment** | **Response** |
| 1 | The recruitment strategy for participants should be elaborated. Details on how participants were selected and the criteria for inclusion would strengthen the protocol. Consider addressing potential barriers to participation and strategies for overcoming them. | Thank you for pointing this out. We have provided more details on the inclusion criteria for participants and on the recruitment strategies we will use in the three phases of the study. We have also highlighted the length of the study as a potential barrier to retaining participants through the phases and we mentioned the close contact between members of the research team as a resource for recruiting new participants. In addition, we discussed providing Amazon gift cards as a strategy to prevent attrition. |
| 2 | Clarifying the specific outcome measures that will be used to assess self-determination would add to the protocol’s robustness. | In the final step of Phase 3 (Planning Evaluation Strategies), we made it clear that the AUTODDIS scale would be used to measure self-determination outcomes of the intervention at the time of its implementation. |
| 3 | The manuscript partially meets the ethics of experimentation and research integrity standards. | In response to reviewer 1’s comment (see comment 5 below), we have clarified in the manuscript the strategies we will use to promote methodological integrity. |
| 4 | By summing up, this protocol holds significant promise for contributing to the field of intellectual disabilities. With major revision addressing recruitment strategy, result analyses, specific outcome measures, and ethics of experimentation, can effectively guide the development and implementation of the valuable intervention. | Thank you for your kind comments on our manuscript. |
| **REVIEWER 1** | | |
| 5 | While this manuscript presents a promising conceptual framework for a study yet to be conducted, several concerns warrant attention, particularly regarding potential biases in participant selection. Theoretical sampling is a reasonable approach; however, to prevent bias, it is essential to implement purposive selection, a theory-driven approach, reflexivity, triangulation, peer debriefing, transparent decision-making, and negative case analysis—all of which are crucial safeguards mentioned to varying degrees in the manuscript. These elements must be rigorously adhered to in practice to ensure methodological integrity. | Thank you for pointing this out. In the previous version of the manuscript, in the “Quality Assurance” section, we explained that some of these strategies would be used, including the use of a reflexive journal to promote reflexivity and the development of an audit trail to promote transparency. We also made clear that criteria specific to grounded theory and concept mapping would be used to ensure quality in the implementation of both approaches. In this resubmitted version, as suggested by the reviewer, we have added three additional strategies: investigator triangulation and negative case analysis during data analysis in phase 1, and peer debriefing in all three phases. In addition, the sampling strategy will be purposive in the three phases and, as explained throughout the protocol, the methodological approach is clearly guided by Causal Agency Theory. |
| 6 | A critical concern is the need for careful consideration of bias in participant selection, especially concerning socioeconomic diversity. The manuscript does not acknowledge the importance of this aspect and should explicitly outline strategies to ensure adequate representation across socioeconomic groups. Ensuring inclusivity in participant selection is crucial for minimizing biases and enhancing the study's applicability and relevance to broader populations. | Thank you for mentioning this. While the aim of a grounded theory is to achieve representational and theoretical generalization rather than population generalization, we agree with the reviewer on the relevance of including a heterogeneous sample in terms of socio-demographic attributes. We have made it clear in the limitations section that we will strive to build a varied sample, but this may not be possible because participation will depend on both the types of families using the services of the associations to be contacted and the willingness of those families to participate in the study. |
| 7 | Another significant concern highlighted is the potential lack of generalizability of the study findings. While grounded theory studies typically aim for theoretical rather than statistical generalizability, the manuscript should address this limitation transparently. Strategies to mitigate this concern could include detailing the contexts in which findings may be applicable or discussing the transferability of insights to similar settings. | In the “Discussion” section, we have described the limitations of the study methods for generalizing the findings. We have also explained that “thick description” (i.e., providing a detailed account of the participants’ views, meanings, and understandings) will help other researchers to assess the transferability of the study findings to other settings and contexts, thereby enhancing the generalizability of the findings. |
| 8 | Another crucial issue requiring attention in the manuscript is how the research team plans to address potential environmental hazards, allegations of physical and mental abuse situations or lack of support services identified during interviews and photo elicitation sessions. | In the Ethics and Dissemination section, we have highlighted that some of the investigators are experienced therapists who can respond to any participant’s needs, concerns or sensitive issues that may arise during the research process. |
| **REVIEWER 2** | | |
| 9 | While the protocol is detailed (perhaps too detailed in parts, particularly in the early stages) the latter stages of the study are not explained to the same extent. The process for conducting Phase 3, in particular, would benefit from further detail. | We appreciate this comment. Phase 3 will build on the findings of the previous two phases, and the final steps of this phase will also depend on the initial steps. This makes it more difficult to define concrete and definitive procedures for this phase. Despite these limitations, we have provided further details about the procedures we will follow in each of the steps. We hope that the explanation of the method is now more precise, but we are eager to provide additional information if needed. |
| 10 | Although a definition of intellectual disability is given in lines 195-196, the manuscript would benefit from an earlier definition bearing in mind that this is not a subject specific journal and many readers may not be familiar with intellectual disability. The term should be defined in the Introduction so that the reader understands the cohort under review from the outset. | We have added to the beginning of the Introduction two widely known definitions of intellectual disability proposed by the American Association on Intellectual and Developmental Disabilities and the American Psychiatric Association. |
| 11 | Although the concept of self-determination is explained in the Introduction, an earlier placement of the definition should be considered. | We have moved the definition of self-determination to the first paragraph, just after the definition of intellectual disability. |
| 12 | Line 88 refers to RQ4 but there is no further reference to this research question in the manuscript. Why is this the case? | Thank you for bringing this to our attention. RQ4 is complementary to RQ3. We have added the mention of RQ 4 in the parts of the manuscript related to the intervention mapping method. |
| 13 | The Theoretical Framework section is rather long and provides much information not directly relevant to this study. It could perhaps be summarised with a focus on why the selected framework is particularly suited to this study. | We agree with this consideration and have significantly shortened this section. |
| 14 | Lines 176-177 mentions that an early literature review is recommended – has this been carried out? | We have made it clear that a nonsystematic literature review was conducted in the early stages of protocol development. |
| 15 | Sampling and recruitment (line 189 on) – this section discusses participating families but it is not clear until further in the section (line 253) who the participant could be and what is meant by ‘family’. This could be explained earlier in the section to make it clearer for the reader. | Just after mentioning that families will be sampled in this study, we have clarified that participants in each family will include one or both parents or the primary caregiver. |
| 16 | Line 272 – data collection in Phase 1 involves a number of interviews and a photo elicitation task. It is not clear if the same person must take part in all of these or whether a different parent could participate? | While the number of participants from each family may vary between interviews (e.g., the second interview may include both the mother and father, while the third interview may include only the mother), at least one participant (i.e., the same person) must participate in all three interviews. We have made this clear in the manuscript. |
| 17 | For Phase 2, will recruitment and data collection and analysis take place concurrently as will occur for Phase 1? | In Phase 2, recruitment will occur prior to the implementation of the first step of the method. As reported in the concept mapping literature, it is likely that this number will decrease in subsequent steps. However, we will strive to have no less than 30-35 participants in these steps (a number consistent with recommendations in the literature), recruiting new participants if necessary. Data analysis will be conducted in step 4 after data collection (steps 1, 2 and 3) has been completed. We have made this clear in the manuscript. |
| 18 | At what point will Phase 2 recruitment conclude? | See response to comment number 17. |
| 19 | The paragraph from line 380 onwards is very long. The authors should consider dividing into separate paragraphs according to each step in the process. | We have shortened the paragraph by splitting it into two paragraphs that refer to step 1 (first paragraph) and to steps 2 and 3 (second paragraph). |
| 20 | Phase 3 – it is not specified how many participants will be required. Will new participants be recruited if necessary? | We have explained that three focus groups of 10 participants each will be held in step 1 and two with ideally the same participants in step 2. Therefore, the total number of participants should be around 30, subject to increase if participants from the first focus group are unavailable for the second. If necessary, new participants with similar characteristics to those in the previous phases will be recruited. |
| 21 | Line 453 mentions that two workshops will be held however detail around these workshops is light and not to the same level as detail provided for data collection in earlier phases of the study. For example, how many participants will be in each workshop, will the workshops be audio recorded and how will the data be analysed? | We agree with this comment. To be more methodologically precise, we have replaced the term “workshop” with “focus groups”. We have also provided more details about the total number of focus group participants (n=30) and the procedures we will use to conduct, transcribe, and analyze the focus group data. |
| 22 | Similarly line 487 mentions two additional workshops but no further detail is given as to the procedure and outputs from these. | We have made it clear that the same sampling, data collection, and analysis procedures from the focus groups conducted in Step 1 will be followed in these additional two focus groups conducted in Step 5. We have also provided more details about the type of information that will be collected in these two focus groups. |
| 23 | Line 539 mentions that participant data will be held on a computer belonging to one of the authors. This raises concerns around data protection eg implications of loss/theft of the computer? Have provisions been made to back up the data? | Thank you for this suggestion. We have added that a backup of the data will be stored on two encrypted external drives that will be accessible only to the researchers. |
| 24 | Line 571 mentions the risk of not meeting the criterion for saturation in Phase 1. What about the risk of not recruiting sufficient participants in other phases? | We explicitly stated that this risk applies to the other phases as well. However, we also mentioned that grounded theory is characterized by stricter sampling criteria than participatory group concept mapping and intervention mapping. |
| 25 | Line 590 mentions the possibility of having to recruit new participants in subsequent phases. Has the impact of including potentially different participants across phases been considered? | In Phase 2, this should not present a significant challenge because the sample size needs to be larger than in Phase 1, thereby requiring the recruitment of new participants. With respect to Phase 3, if participants from Phases 1 and 2 do not agree to participate or are unavailable, we will recruit new individuals who meet our inclusion criteria and have similar characteristics to those in the previous phases. We have made this clear in the manuscript text. |
| 26 | In keeping with current protocol, it would be preferable to use the full term for intellectual disability throughout the paper rather than an abbreviation | We have replaced abbreviations of terms throughout the manuscript, including those related to intellectual disability, research questions, grounded theory, participatory group concept mapping, and intervention mapping, with the full term. |
| 27 | The format for in-text referencing (where authors are named) should be reviewed to ensure adherence to standards. | In-text referencing has been revised to ensure compliance with the Verdana referencing style. We used EndNote to insert the references into the manuscript. |
