# Supplementary material for: Development of a family-centered intervention to support self-determination in adolescents and young adults with intellectual disability in home environments: Protocol for a multistage mixed methods design
Source: PLoS One. 2025 Sep 4;20(9):e0325919. doi: 10.1371/journal.pone.0325919 (PMC12410785; doi:10.1371/journal.pone.0325919)
Supplement: Attachment — Submitted filename: Response_to_Reviewers_auresp_2.docx [file pone.0325919.s002.docx]

Hina Hadayat Ali, PhD

Academic Editor, PLOS ONE

Dear Dr. Ali,

We would like to thank you and the reviewers for your feedback on our manuscript entitled “Development of a family-centered intervention to support self-determination in adolescents and young adults with intellectual disability in home environments: Protocol for a multistage mixed methods design” and for the opportunity to submit a second revised version of the manuscript. As requested, we have revised the manuscript based on reviewer 2’s suggestions, and we believe that these revisions have improved the overall quality of the manuscript. To facilitate review, we have prepared a table detailing our revisions. We have also used the Track Changes feature in Word to indicate the changes we have made to the manuscript.

| **EDITOR** | | |
| --- | --- | --- |
|  | **Comment** | **Response** |
| 1 | There is concern about the lack of specific strategies to ensure representation across socioeconomic groups, even though the authors aim for a varied sample. The limitation of the study’s findings being applicable only to participants similar to those in the study is viewed as a significant drawback to an otherwise robust design. | Thank you for this note. See our response to the reviewer below. |
| **REVIEWER 2** | | |
| 2 | While most of the earlier review comments have been addressed, I feel that concerns remain about the potential for bias in participant selection. Though the authors have responded that they will strive to build a varied sample, there are no specific strategies outlined which will seek to ensure adequate representation across socioeconomic groups (…) Could greater efforts not be made to target particular groups and these strategies described? For example, could the institutions and special education schools contacted not be drawn from a cross-section of socioeconomic settings to ensure there is some degree of representativeness? | Thank you for this comment. In the Sampling and Recruitment subsection, we explained that we will aim to achieve socioeconomic diversity using the stratified random sampling strategy as suggested by Patton (2014). In line with this strategy, when identifying families to be recruited, we will select institutions and special education schools from different socioeconomic areas using socioeconomic indices in each region, if available. We agree with the reviewer that such a strategy will help to broaden the scope of the intervention to be developed in phase 3 of the study, particularly in terms of the population to which the intervention is applicable. Accordingly, we have removed the limitation that the results will be only applicable to a specific profile of individuals. |
